# Supplementary material for: Pyruvate Dehydrogenase Kinase 1 inhibition mediated oxidative phosphorylation enhancement in cartilage promotes osteoarthritis progression
Source: BMC Musculoskelet Disord. 2023 Jul 20;24:597. doi: 10.1186/s12891-023-06585-6 (PMC10357736; doi:10.1186/s12891-023-06585-6)
Supplement: Supplementary file 1 — Additional file 1. [file 12891_2023_6585_MOESM1_ESM.zip › KEGG.Gsea.1653622432464/KEGG_ERBB_SIGNALING_PATHWAY.html]

Details for gene set KEGG\_ERBB\_SIGNALING\_PATHWAY[GSEA]

|  || Dataset | 55457\_collapsed\_to\_symbols.cls.cls #OA\_versus\_normal.cls.cls #OA\_versus\_normal\_repos |
| Phenotype | cls.cls#OA\_versus\_normal\_repos |
| Upregulated in class | normal |
| GeneSet | KEGG\_ERBB\_SIGNALING\_PATHWAY |
| Enrichment Score (ES) | -0.29025742 |
| Normalized Enrichment Score (NES) | -1.2145578 |
| Nominal p-value | 0.15930599 |
| FDR q-value | 0.35260004 |
| FWER p-Value | 1.0 |
Table: GSEA Results Summary

  

Fig 1: Enrichment plot: KEGG\_ERBB\_SIGNALING\_PATHWAY      
 Profile of the Running ES Score & Positions of GeneSet Members on the Rank Ordered List

  

| SYMBOL | TITLE | RANK IN GENE LIST | RANK METRIC SCORE | RUNNING ES | CORE ENRICHMENT || 1 | NCK1 | NCK adaptor protein 1 [Source:HGNC Symbol;Acc:HGNC:7664] | 222 | 0.804 | 0.0130 | No |
| 2 | MAPK9 | mitogen-activated protein kinase 9 [Source:HGNC Symbol;Acc:HGNC:6886] | 721 | 0.588 | -0.0037 | No |
| 3 | PLCG2 | phospholipase C gamma 2 [Source:HGNC Symbol;Acc:HGNC:9066] | 725 | 0.588 | 0.0182 | No |
| 4 | MAPK3 | mitogen-activated protein kinase 3 [Source:HGNC Symbol;Acc:HGNC:6877] | 757 | 0.579 | 0.0376 | No |
| 5 | CAMK2A | calcium/calmodulin dependent protein kinase II alpha [Source:HGNC Symbol;Acc:HGNC:1460] | 916 | 0.538 | 0.0456 | No |
| 6 | ERBB2 | erb-b2 receptor tyrosine kinase 2 [Source:HGNC Symbol;Acc:HGNC:3430] | 963 | 0.526 | 0.0618 | No |
| 7 | AKT1 | AKT serine/threonine kinase 1 [Source:HGNC Symbol;Acc:HGNC:391] | 1192 | 0.478 | 0.0621 | No |
| 8 | CRK | "CRK proto-oncogene, adaptor protein [Source:HGNC Symbol;Acc:HGNC:2362]" | 2018 | 0.360 | 0.0113 | No |
| 9 | PIK3CB | "phosphatidylinositol-4,5-bisphosphate 3-kinase catalytic subunit beta [Source:HGNC Symbol;Acc:HGNC:8976]" | 2186 | 0.340 | 0.0111 | No |
| 10 | MAP2K4 | mitogen-activated protein kinase kinase 4 [Source:HGNC Symbol;Acc:HGNC:6844] | 2214 | 0.337 | 0.0216 | No |
| 11 | CRKL | "CRK like proto-oncogene, adaptor protein [Source:HGNC Symbol;Acc:HGNC:2363]" | 2283 | 0.329 | 0.0287 | No |
| 12 | EGF | epidermal growth factor [Source:HGNC Symbol;Acc:HGNC:3229] | 2399 | 0.315 | 0.0316 | No |
| 13 | SHC1 | SHC adaptor protein 1 [Source:HGNC Symbol;Acc:HGNC:10840] | 2485 | 0.304 | 0.0365 | No |
| 14 | CAMK2G | calcium/calmodulin dependent protein kinase II gamma [Source:HGNC Symbol;Acc:HGNC:1463] | 2900 | 0.261 | 0.0140 | No |
| 15 | CDKN1B | cyclin dependent kinase inhibitor 1B [Source:HGNC Symbol;Acc:HGNC:1785] | 2984 | 0.251 | 0.0170 | No |
| 16 | BTC | betacellulin [Source:HGNC Symbol;Acc:HGNC:1121] | 2997 | 0.250 | 0.0255 | No |
| 17 | ELK1 | ETS transcription factor ELK1 [Source:HGNC Symbol;Acc:HGNC:3321] | 3357 | 0.216 | 0.0056 | No |
| 18 | PRKCA | protein kinase C alpha [Source:HGNC Symbol;Acc:HGNC:9393] | 3728 | 0.183 | -0.0164 | No |
| 19 | BAD | BCL2 associated agonist of cell death [Source:HGNC Symbol;Acc:HGNC:936] | 3788 | 0.178 | -0.0142 | No |
| 20 | PAK1 | p21 (RAC1) activated kinase 1 [Source:HGNC Symbol;Acc:HGNC:8590] | 3861 | 0.169 | -0.0135 | No |
| 21 | NRG2 | neuregulin 2 [Source:HGNC Symbol;Acc:HGNC:7998] | 4510 | 0.112 | -0.0598 | No |
| 22 | ERBB3 | erb-b2 receptor tyrosine kinase 3 [Source:HGNC Symbol;Acc:HGNC:3431] | 4863 | 0.083 | -0.0842 | No |
| 23 | RPS6KB2 | ribosomal protein S6 kinase B2 [Source:HGNC Symbol;Acc:HGNC:10437] | 5496 | 0.033 | -0.1322 | No |
| 24 | STAT5B | signal transducer and activator of transcription 5B [Source:HGNC Symbol;Acc:HGNC:11367] | 5554 | 0.028 | -0.1356 | No |
| 25 | GRB2 | growth factor receptor bound protein 2 [Source:HGNC Symbol;Acc:HGNC:4566] | 5697 | 0.016 | -0.1461 | No |
| 26 | SHC2 | SHC adaptor protein 2 [Source:HGNC Symbol;Acc:HGNC:29869] | 5738 | 0.012 | -0.1487 | No |
| 27 | EIF4EBP1 | eukaryotic translation initiation factor 4E binding protein 1 [Source:HGNC Symbol;Acc:HGNC:3288] | 5871 | 0.004 | -0.1589 | No |
| 28 | RAF1 | "Raf-1 proto-oncogene, serine/threonine kinase [Source:HGNC Symbol;Acc:HGNC:9829]" | 6187 | -0.019 | -0.1827 | No |
| 29 | HBEGF | heparin binding EGF like growth factor [Source:HGNC Symbol;Acc:HGNC:3059] | 6409 | -0.036 | -0.1986 | No |
| 30 | PRKCB | protein kinase C beta [Source:HGNC Symbol;Acc:HGNC:9395] | 6453 | -0.039 | -0.2005 | No |
| 31 | ERBB4 | erb-b2 receptor tyrosine kinase 4 [Source:HGNC Symbol;Acc:HGNC:3432] | 6830 | -0.065 | -0.2274 | No |
| 32 | MAPK1 | mitogen-activated protein kinase 1 [Source:HGNC Symbol;Acc:HGNC:6871] | 6853 | -0.068 | -0.2266 | No |
| 33 | SRC | "SRC proto-oncogene, non-receptor tyrosine kinase [Source:HGNC Symbol;Acc:HGNC:11283]" | 6940 | -0.075 | -0.2304 | No |
| 34 | STAT5A | signal transducer and activator of transcription 5A [Source:HGNC Symbol;Acc:HGNC:11366] | 6988 | -0.079 | -0.2311 | No |
| 35 | NRAS | "NRAS proto-oncogene, GTPase [Source:HGNC Symbol;Acc:HGNC:7989]" | 7300 | -0.102 | -0.2515 | No |
| 36 | PTK2 | protein tyrosine kinase 2 [Source:HGNC Symbol;Acc:HGNC:9611] | 7315 | -0.103 | -0.2487 | No |
| 37 | PIK3CD | "phosphatidylinositol-4,5-bisphosphate 3-kinase catalytic subunit delta [Source:HGNC Symbol;Acc:HGNC:8977]" | 7405 | -0.111 | -0.2515 | No |
| 38 | PAK5 | p21 (RAC1) activated kinase 5 [Source:HGNC Symbol;Acc:HGNC:15916] | 7558 | -0.124 | -0.2587 | No |
| 39 | PIK3CA | "phosphatidylinositol-4,5-bisphosphate 3-kinase catalytic subunit alpha [Source:HGNC Symbol;Acc:HGNC:8975]" | 7733 | -0.136 | -0.2671 | No |
| 40 | PAK2 | p21 (RAC1) activated kinase 2 [Source:HGNC Symbol;Acc:HGNC:8591] | 7762 | -0.139 | -0.2641 | No |
| 41 | PIK3CG | "phosphatidylinositol-4,5-bisphosphate 3-kinase catalytic subunit gamma [Source:HGNC Symbol;Acc:HGNC:8978]" | 7953 | -0.152 | -0.2732 | No |
| 42 | HRAS | "HRas proto-oncogene, GTPase [Source:HGNC Symbol;Acc:HGNC:5173]" | 7977 | -0.153 | -0.2692 | No |
| 43 | AKT3 | AKT serine/threonine kinase 3 [Source:HGNC Symbol;Acc:HGNC:393] | 8008 | -0.156 | -0.2657 | No |
| 44 | SOS2 | SOS Ras/Rho guanine nucleotide exchange factor 2 [Source:HGNC Symbol;Acc:HGNC:11188] | 8058 | -0.160 | -0.2635 | No |
| 45 | ARAF | "A-Raf proto-oncogene, serine/threonine kinase [Source:HGNC Symbol;Acc:HGNC:646]" | 8092 | -0.163 | -0.2599 | No |
| 46 | BRAF | "B-Raf proto-oncogene, serine/threonine kinase [Source:HGNC Symbol;Acc:HGNC:1097]" | 8171 | -0.169 | -0.2597 | No |
| 47 | PAK3 | p21 (RAC1) activated kinase 3 [Source:HGNC Symbol;Acc:HGNC:8592] | 8266 | -0.176 | -0.2603 | No |
| 48 | PRKCG | protein kinase C gamma [Source:HGNC Symbol;Acc:HGNC:9402] | 8582 | -0.200 | -0.2774 | No |
| 49 | TGFA | transforming growth factor alpha [Source:HGNC Symbol;Acc:HGNC:11765] | 8748 | -0.212 | -0.2823 | Yes |
| 50 | CAMK2B | calcium/calmodulin dependent protein kinase II beta [Source:HGNC Symbol;Acc:HGNC:1461] | 8782 | -0.215 | -0.2767 | Yes |
| 51 | GAB1 | GRB2 associated binding protein 1 [Source:HGNC Symbol;Acc:HGNC:4066] | 8786 | -0.215 | -0.2688 | Yes |
| 52 | PIK3R3 | phosphoinositide-3-kinase regulatory subunit 3 [Source:HGNC Symbol;Acc:HGNC:8981] | 8801 | -0.217 | -0.2618 | Yes |
| 53 | PIK3R5 | phosphoinositide-3-kinase regulatory subunit 5 [Source:HGNC Symbol;Acc:HGNC:30035] | 8804 | -0.217 | -0.2537 | Yes |
| 54 | MAPK8 | mitogen-activated protein kinase 8 [Source:HGNC Symbol;Acc:HGNC:6881] | 8956 | -0.228 | -0.2569 | Yes |
| 55 | PAK6 | p21 (RAC1) activated kinase 6 [Source:HGNC Symbol;Acc:HGNC:16061] | 9068 | -0.237 | -0.2566 | Yes |
| 56 | ABL1 | "ABL proto-oncogene 1, non-receptor tyrosine kinase [Source:HGNC Symbol;Acc:HGNC:76]" | 9081 | -0.238 | -0.2486 | Yes |
| 57 | NRG1 | neuregulin 1 [Source:HGNC Symbol;Acc:HGNC:7997] | 9209 | -0.248 | -0.2491 | Yes |
| 58 | CBL | Cbl proto-oncogene [Source:HGNC Symbol;Acc:HGNC:1541] | 9479 | -0.272 | -0.2599 | Yes |
| 59 | CBLC | Cbl proto-oncogene C [Source:HGNC Symbol;Acc:HGNC:15961] | 9553 | -0.279 | -0.2550 | Yes |
| 60 | SHC3 | SHC adaptor protein 3 [Source:HGNC Symbol;Acc:HGNC:18181] | 9608 | -0.284 | -0.2486 | Yes |
| 61 | MAPK10 | mitogen-activated protein kinase 10 [Source:HGNC Symbol;Acc:HGNC:6872] | 9699 | -0.291 | -0.2446 | Yes |
| 62 | NCK2 | NCK adaptor protein 2 [Source:HGNC Symbol;Acc:HGNC:7665] | 9755 | -0.297 | -0.2377 | Yes |
| 63 | MAP2K2 | mitogen-activated protein kinase kinase 2 [Source:HGNC Symbol;Acc:HGNC:6842] | 9932 | -0.315 | -0.2396 | Yes |
| 64 | PAK4 | p21 (RAC1) activated kinase 4 [Source:HGNC Symbol;Acc:HGNC:16059] | 10004 | -0.322 | -0.2330 | Yes |
| 65 | MAP2K1 | mitogen-activated protein kinase kinase 1 [Source:HGNC Symbol;Acc:HGNC:6840] | 10197 | -0.340 | -0.2351 | Yes |
| 66 | RPS6KB1 | ribosomal protein S6 kinase B1 [Source:HGNC Symbol;Acc:HGNC:10436] | 10224 | -0.343 | -0.2242 | Yes |
| 67 | KRAS | "KRAS proto-oncogene, GTPase [Source:HGNC Symbol;Acc:HGNC:6407]" | 10544 | -0.377 | -0.2349 | Yes |
| 68 | PLCG1 | phospholipase C gamma 1 [Source:HGNC Symbol;Acc:HGNC:9065] | 10722 | -0.395 | -0.2338 | Yes |
| 69 | EREG | epiregulin [Source:HGNC Symbol;Acc:HGNC:3443] | 10778 | -0.403 | -0.2229 | Yes |
| 70 | PIK3R2 | phosphoinositide-3-kinase regulatory subunit 2 [Source:HGNC Symbol;Acc:HGNC:8980] | 10956 | -0.425 | -0.2206 | Yes |
| 71 | PIK3R1 | phosphoinositide-3-kinase regulatory subunit 1 [Source:HGNC Symbol;Acc:HGNC:8979] | 11099 | -0.443 | -0.2150 | Yes |
| 72 | GSK3B | glycogen synthase kinase 3 beta [Source:HGNC Symbol;Acc:HGNC:4617] | 11157 | -0.451 | -0.2025 | Yes |
| 73 | SOS1 | SOS Ras/Rac guanine nucleotide exchange factor 1 [Source:HGNC Symbol;Acc:HGNC:11187] | 12041 | -0.603 | -0.2486 | Yes |
| 74 | MTOR | mechanistic target of rapamycin kinase [Source:HGNC Symbol;Acc:HGNC:3942] | 12047 | -0.605 | -0.2262 | Yes |
| 75 | EGFR | epidermal growth factor receptor [Source:HGNC Symbol;Acc:HGNC:3236] | 12109 | -0.622 | -0.2075 | Yes |
| 76 | AREG | amphiregulin [Source:HGNC Symbol;Acc:HGNC:651] | 12190 | -0.641 | -0.1895 | Yes |
| 77 | MAP2K7 | mitogen-activated protein kinase kinase 7 [Source:HGNC Symbol;Acc:HGNC:6847] | 12209 | -0.649 | -0.1665 | Yes |
| 78 | ABL2 | "ABL proto-oncogene 2, non-receptor tyrosine kinase [Source:HGNC Symbol;Acc:HGNC:77]" | 12342 | -0.688 | -0.1508 | Yes |
| 79 | CBLB | Cbl proto-oncogene B [Source:HGNC Symbol;Acc:HGNC:1542] | 12725 | -0.887 | -0.1472 | Yes |
| 80 | CDKN1A | cyclin dependent kinase inhibitor 1A [Source:HGNC Symbol;Acc:HGNC:1784] | 12727 | -0.890 | -0.1137 | Yes |
| 81 | MYC | "MYC proto-oncogene, bHLH transcription factor [Source:HGNC Symbol;Acc:HGNC:7553]" | 12801 | -0.970 | -0.0828 | Yes |
| 82 | JUN | "Jun proto-oncogene, AP-1 transcription factor subunit [Source:HGNC Symbol;Acc:HGNC:6204]" | 12813 | -0.994 | -0.0462 | Yes |
| 83 | AKT2 | AKT serine/threonine kinase 2 [Source:HGNC Symbol;Acc:HGNC:392] | 12896 | -1.404 | 0.0003 | Yes |
Table: GSEA details [plain text format]

  

Fig 2: KEGG\_ERBB\_SIGNALING\_PATHWAY      
 Blue-Pink O' Gram in the Space of the Analyzed GeneSet

  

Fig 3: KEGG\_ERBB\_SIGNALING\_PATHWAY: Random ES distribution      
 Gene set null distribution of ES for **KEGG\_ERBB\_SIGNALING\_PATHWAY**
